# Epidemiology of ventilator associated pneumonia (vap) in patients with abdominal sepsis

**DOI:** 10.1186/2197-425X-3-S1-A280

**Published:** 2015-10-01

**Authors:** A Hom Choudhuri, U Bhatia Batra, P Harisinghani, R Uppal

**Affiliations:** GB Pant Institute of Post Graduate Medical Education & Research, Anaesthesiology & Intensive Care, New Delhi, India

## Introduction

Abdominal infection leading to sepsis is a major cause of mortality in the ICU. The inflammatory response also alters the pulmonary immunity leading to increased risk for VAP.

## Objectives

The aim of this study was to determine the incidence of VAP and define the most important pathogens for VAP in patients with abdominal sepsis.

## Methods

A retrospective study in a 7 bedded mixed medical surgical ICU of a tertiary care teaching institute.

The data of patients diagnosed with abdominal sepsis between 2009 & 2013 and requiring mechanical ventilation for at least 48 hours was extracted. The microbiological flora as assessed by sampling of endotracheal aspirate (ETA) for culture and sensitivity were noted. The Clinical Pulmonary Infection Score (CPIS) was used to make the diagnosis of VAP.

## Results

The average age of the 124 patients (59 males and 65 females) recruited in the study was 58 years (range 27-76 years) and mean APACHE II was 19 (range 9-30). The average length of ICU stay was 16 days (range 4-57 days) and the duration of mechanical ventilation was 12.5 days; 39 patients (31.4%) died. In patients with abdominal sepsis, 67 strains of bacteria were cultivated from the lower respiratory tract, of which 62 were Gram-negative. All of these bacterial strains were isolated in significant quantities for lower respiratory tract infection. The most common bacteria isolated from the endotracheal aspirate were *Klebsiella pneumoniae* (24 times), followed by *Pseudomonas aeruginosa* (22 times) and *Acinetobacter baumannii* (16 times). VAP was diagnosed in 47 patients (37.9%) in the cohort. The incidence of VAP was calculated to be 93.6 per 1000 days of mechanical ventilation. In 34 patients (72.3%), VAP was diagnosed as early onset ( < 4 days of initiation of mechanical ventilation) and in 13 patients (27.7%) as late onset (>4 days).

## Conclusions

Despite advances in diagnostics and therapy, abdominal sepsis is still burdened with high morbidity and mortality due to VAP. Therefore, the prevention and treatment of VAP remains an integral component in the management of mechanically ventilated patients of abdominal sepsis.
